# Neural networks and particle swarm for transformer oil diagnosis by dissolved gas analysis

**DOI:** 10.1038/s41598-024-60071-0

**Published:** 2024-04-23

**Authors:** Fettouma Guerbas, Youcef Benmahamed, Youcef Teguar, Rayane Amine Dahmani, Madjid Teguar, Enas Ali, Mohit Bajaj, Shir Ahmad Dost Mohammadi, Sherif S. M. Ghoneim

**Affiliations:** 1 Laboratoire des Systèmes Electriques et Industriels, Université des Sciences et de la Technologie Houari Boumediene, BP 32Bab Ezzouar, 16111 Algiers, Algeria; 2 Laboratoire de Recherche en Electrotechnique, Ecole Nationale Polytechnique, 10 Rue des Frères Oudek, El Harrach, 16200 Algiers, Algeria; 3 Centre of Research Impact and Outcome, Chitkara University Institute of Engineering and Technology, Chitkara University, Rajpura, Punjab 140401 India; 4 Department of Electrical Engineering, Graphic Era (Deemed to Be University), Dehradun, 248002 India; 5 Hourani Center for Applied Scientific Research, Al-Ahliyya Amman University, Amman, Jordan; 6 Graphic Era Hill University, Dehradun, 248002 India; 7 Department of Electrical and Electronics, Faculty of Engineering, Alberoni University, Kapisa, Afghanistan; 8 Department of Electrical Engineering, College of Engineering, Taif University, P.O. BOX 11099, 21944 Taif, Saudi Arabia

**Keywords:** Power transformer, Insulating oil, Diagnosis, Dissolved gas analysis, Electrical and thermal faults, Artificial neural networks, Particle swarm algorithm, Optimization, Energy science and technology, Engineering, Mathematics and computing

## Abstract

The lifetime of power transformers is closely related to the insulating oil performance. This latter can degrade according to overheating, electric arcs, low or high energy discharges, etc. Such degradation can lead to transformer failures or breakdowns. Early detection of these problems is one of the most important steps to avoid such failures. More efficient diagnostic systems, such as artificial intelligence techniques, are recommended to overcome the limitations of the classical methods. This work deals with diagnosing the power transformer insulating oil by analysis of dissolved gases using new techniques. For this, we have proposed intelligent techniques based on Multilayer artificial neural networks (ANN). Thus, a multi-layer ANN-based model for fault detection is presented. To improve its classification rate, this one was optimized by a meta-heuristic technique as the particle swarm optimization (PSO) technique. Optimized ANNs have never been used in transformer insulating oil diagnostics so far. The robustness and effectiveness of the proposed model is demonstrated, and high accuracy is obtained.

## Introduction

Power transformers are crucial and essential components in the electrical power transmission and distribution networks. Such expensive electrical devices should work properly for years^1^. The lifetime of a power transformer closely depends on its insulation system, generally consisting of a traditional solid component (paper, etc.) and a dielectric fluid^2^.

Most power transformers use insulating oil as dielectric fluid, due to its low price and good physico-chemical properties. Besides insulation, this oil dissipates the heat generated by the magnetic circuit and the windings. Following its movement in a transformer in service, the insulating oil conducts this heat to the internal cooling systems (radiators, etc.), before releasing it into the environment^2,3^.

Insulating oil is subjected to several electrical, thermal and chemical constraints in service. These latter lead to the gradual degradation of the insulating oil and eventually cause the transformer to de-energize when not analyzed in time^4,5^. Indeed, various oil analyses are proposed to diagnose the power transformer's internal state. The most popular are physico-chemical analyzes^2–4^ and dissolved gas analysis (DGA)^5–10^.

DGA is a widely used as diagnostic technique. It is based on interpreting the concentrations of gases dissolved in the insulating oil. Indeed, the oil decomposes under electrical and thermal stresses, releasing gases in small quantities^5–10^. In fact, DGA can be performed by introducing sensors into the transformers in service (online mode), or in the laboratory on samples (offline mode)^8^.

The five main gases resulting from the oil decomposition are hydrogen (H_2_), methane (CH_4_), acetylene (C_2_H_2_), ethylene (C_2_H_4_) and ethane (C_2_H_6_). The proportions of the concentrations of these gases in a sample allow determining the defect type^5–10^. According to IEC 60599 (2007)^11^ and IEEE Standard C57.104^12^, six electrical and thermal faults exist. They consist of partial discharges (PD), low energy discharges (D1), high energy discharges (D2), thermal faults for T < 300 °C (T1), thermal faults for T from 300 °C to 700 °C (T2) and, finally, thermal faults for T > 700 °C (T3).

Various traditional techniques have been developed to interpret the results of DGA of transformer oil^13,14^. The most popular use gas concentration ratios of Dornenburg^15^, Rogers^16^, and IEC 60599 (1978)^17^, or graphical methods of Duval employing percentages of concentration ratios such as the triangle^18^ and the pentagon^19^. Although these techniques are simple and easy to implement, they have some drawbacks. First, they use only specific gas ratios. Their accuracy remains limited and are very sensitive to DGA data uncertainties. For instance, Duval’s triangle method showed certain PD detection failures and some interferences between thermal and electrical faults. IEC 60,599 technique presented some interferences between D1 and D2 faults. Except T1, the effectiveness of Rogers' methods has not been demonstrated for the other faults^6^. Finally, Dornenburg's method considers only three faults: thermal decomposition, partial discharges or low energy corona, and high energy electric arcs^15^.

Recent artificial intelligence and meta-heuristic approaches have been integrated with traditional methods to overcome such difficulties and improve the transformer oil diagnostic by DGA. Benmahamed et al. have developed two algorithms to improve the classification rate of the Duval pentagon (initially at 80%). The first (Duval pentagon-SVM-PSO) combines the Duval pentagon and support vector machines (SVM), whose parameters have been optimized by the particle swarm optimization technique, PSO. The second (Duval pentagon-KNN) combines Duval pentagon and the K-nearest neighbors (kNN) algorithm.

The accuracy rate of the first algorithm is 88% compared to 82% for the second^20^. In another research work, Benmahamed et al. established two classifiers KNN and Naïve Bayes (NB) to diagnose transformer oil by DGA. The KNN algorithm provided the highest accuracy rate of 92%^21^. Furthermore, Benmahamed et al. developed two classifiers. The first is Gaussian and the second (SVM-Bat) uses Support Vector Machines (SVM), whose parameters have been optimized by the Bat algorithm. The SVM-Bat accuracy rate is 93.75% against 69.37% for the Gaussian^5^. Kherif et al. developed an algorithm combining KNN with the decision tree principle. An accuracy rate exceeding 93% was obtained, demonstrating the effectiveness of the proposed algorithm^7^. Taha and al proposed an approach using the particle swarm optimization and the fuzzy-logic (PSO-FS) to enhance the of Rogers’ four-ratio diagnostic accuracy from 47.19 to 85.65% and IEC 60,599 one from 55.09 to 85.03%^22^. Ghoneim et al. established an efficient teaching–learning based optimization (TLBO) a model to optimize both gas concentration percentages and ratios. The proposed algorithm allowed obtaining higher diagnostic accuracy (of 82.02%) than the best (78.65%) offered by other DGA techniques presented in the same paper^23^. Ghoneim et al. developed a smart fault diagnostic approach (SFDA) integrating Dornenburg, Rogers three and four-ratio, IEC three-ratio and Duval triangle techniques. Using gas concentrations, the SFDA algorithm has been improved by ANN. The SFDA algorithm allowed obtaining 79.6%. This accuracy rate has been improved to 87.8% with the integration of ANN^24^. It is worth noting that optimized ANNs have never been used in transformer insulating oil diagnostic so far.

In order to improve the accuracy rate of faults detection in power transformers oil by DGA, multilayer artificial neural networks (ANN) are developed, in this investigation. We opt for a multilayer neural network (MLP) comprising an input layer, two hidden layers and an output. Several input vectors are tested, namely the five gases in ppm and in percentage, Dornenberg ratio, Rogers four-ratio and IEC 60,599 three-ratio as well as the combination between the ratios of Rogers and those of Dornenberg, the centers of mass of the triangle and pentagon of Duval, as well as their combination. Various learning algorithms and activation functions are also considered.

To further improve diagnostic rates, neural networks are optimized using the particle swarm technique (PSO). Indeed, different population sizes have been adopted. The performances of these neural networks have been studied in terms of accuracy rate. A total of 481 sample datasets are considered^5^. Two-thirds are used for the training process (so 321 samples), and the rest (160 samples) for the test. The six fault classes (PD, D1, D2, T1, T2, and T3) recommended by IEC 60599 (2007)^11^ and IEEE Standard C57.104^12^ are adopted. A comparative study is carried out between the different neural networks developed.

In this paper, we have demonstrated that the combination of the artificial neural network and the particle swarm optimization leads to global model. This one takes into account classification problem by learning and also optimization by the PSO. It gives significant results versus to the classical methods and we obtain a high level of accuracy.

## ANN models for insulating oil diagnostic by DGA

The state of the power insulation system is responsible for determining the lifetime of the transformers. This insulation system is generally exposed to some constraints resulting from overheating, carbonization of the paper, electric arcs and low or high energy discharges. Such faults can accelerate insulation degradation, affecting the transformer's reliability and lifetime. Indeed, early detection of these faults can prevent undesirable abnormal operating conditions or failures of power transformers. The dissolved gas analysis (DGA) technique is considered to be one of the fastest and most economical techniques widely used to diagnose power transformer fault types^13^.

As mentioned above, traditional fault diagnostic techniques in power transformers have generally shown their limitations and inconsistencies. Despite their simplicity, these techniques are not really adopted by the scientific community, due to their low accuracy rate in faults detection, except Duval pentagon method giving acceptable rate faults classification^6,20^. That is why artificial intelligence (AI) and/or meta-heuristic approaches can be combined with conventional ones to improve the diagnostic accuracy of power transformer insulating oil further.

This section proposes several artificial neural networks to detect faults in an oil-immersed power transformer. These networks have the same architecture (structure) consisting of a multilayer perceptron (MLP). However, their training algorithms and activation functions are different.

The artificial neural network connection is multilayer and consists of an input, output, and two hidden internal layers. In this structure, neurons belonging to the same layer are not connected. Each layer receives signals from the previous layer and transmits its processing result to the next layer. Thus, the information flows in a one direction, from the input to the output through the hidden layers. Such connection type is called feed-forward artificial neural networks (FFANNs)^25^.

The number of neurons in the input layer is equal to the number of elements of the input vector denoted B. Nine models with input vectors have been considered, namely:

- **1st Model:** The database comprises the concentrations of the five gases in ppm. In such conditions, the input vector of this model is given by: B = [H_2_ CH_4_ C_2_H_2_ C_2_H_4_ C_2_H_6_]^T^
- **2nd Model:** For the same database, the input vector of this model for each sample can be written in terms in gas concentrations in percentages as follows: B = [%H_2_%CH_4_%C_2_H_2_%C_2_H_4_%C_2_H_6_]^T^, with:
  1 $$\% {\text{H}}_{{2}} {\text{ = H}}_{{2}} /\left( {{\text{H}}_{{2}} + {\text{CH}}_{{4}} + {\text{C}}_{{2}} {\text{H}}_{{2}} + {\text{C}}_{{2}} {\text{H}}_{{4}} + {\text{C}}_{{2}} {\text{H}}_{{6}} } \right) \, \times { 1}00$$
  2 $$\% {\text{CH}}_{{4}} = {\text{ CH}}_{{4}} /\left( {{\text{H}}_{{2}} + {\text{CH}}_{{4}} + {\text{C}}_{{2}} {\text{H}}_{{2}} + {\text{C}}_{{2}} {\text{H}}_{{4}} + {\text{C}}_{{2}} {\text{H}}_{{6}} } \right) \, \times { 1}00$$
  3 $$\% {\text{C}}_{{2}} {\text{H}}_{{2}} = {\text{ C}}_{{2}} {\text{H}}_{{2}} /\left( {{\text{H}}_{{2}} + {\text{CH}}_{{4}} + {\text{C}}_{{2}} {\text{H}}_{{2}} + {\text{C}}_{{2}} {\text{H}}_{{4}} + {\text{C}}_{{2}} {\text{H}}_{{6}} } \right) \, \times { 1}00$$
  4 $$\% {\text{C}}_{{2}} {\text{H}}_{{4}} = {\text{ C}}_{{2}} {\text{H}}_{{4}} /\left( {{\text{H}}_{{2}} + {\text{CH}}_{{4}} + {\text{C}}_{{2}} {\text{H}}_{{2}} + {\text{C}}_{{2}} {\text{H}}_{{4}} + {\text{C}}_{{2}} {\text{H}}_{{6}} } \right) \, \times { 1}00$$
  5 $$\% {\text{C}}_{{2}} {\text{H}}_{{6}} = {\text{ C}}_{{2}} {\text{H}}_{{6}} /\left( {{\text{H}}_{{2}} + {\text{CH}}_{{4}} + {\text{C}}_{{2}} {\text{H}}_{{2}} + {\text{C}}_{{2}} {\text{H}}_{{4}} + {\text{C}}_{{2}} {\text{H}}_{{6}} } \right) \, \times { 1}00$$
- **3rd Model:** Dornenburg ratios is one of the first techniques introduced for the power transformer oil diagnostic to interpret the results of dissolved gas analyzes^15^. In this model, we use four ratios of gas, in ppm, consisting of B = [CH_4_/H_2_ C_2_H_2_/C_2_H_4_ C_2_H_4_/C_2_H_6_ C_2_H_2_/CH_4_]^T^
- **4th Model:** The four ratios of Rogers are also considered. For this model, the input vector can be expressed, for each sample (gas in ppm), by Ref.^16^ A = [CH_4_/H_2_ C_2_H_2_/C_2_H_4_ C_2_H_4_/C_2_H_6_ C_2_H_6_/CH_4_]
- **5th Model:** IEC 60,599 model uses three ratios of gas in ppm as follows^17^:
  $${\text{B }} = [{\text{CH}}_{{4}} /{\text{H}}_{{2}} {\text{C}}_{{2}} {\text{H}}_{{2}} /{\text{C}}_{{2}} {\text{H}}_{{4}} {\text{C}}_{{2}} {\text{H}}_{{4}} /{\text{C}}_{{2}} {\text{H}}_{{6}} ]^{{\text{T}}}$$
- **6th Model:** Duval triangle model is a graphic representation using the percentage ratios of three dissolved gases: CH_4_, C_2_H_2_ and C_2_H_4_. These percentages are employed as coordinates (T_x_, T_y_) to draw the mass center point in the triangle and identify, for each sample, the defect zone in which it is located^18^. The input vector for this model is therefore written by: B = [T_x_ T_y_]^T^
- **7th Model:** Duval pentagon model is a graphic representation similar to triangle one. Pentagon uses the five dissolved gas in percentages (%H_2_%CH_4_%C_2_H_2_%C_2_H_4_%C_2_H_6_) to draw the mass center point coordinates (P_x_, P_y_)^19^. The input vector of this model is given by: B = [P_x_ P_y_]^T^
- **8th Model:** A combination between the triangle and the pentagon of Duval was proposed for this model. In such conditions, the two mass centers coordinate of both triangle and pentagon of Duval will constitute the input vector, given by: B = [C_x_ C_y_ P_x_ P_y_]^T^
- **9th Model:** We suggest here another model consisting of combination between Rogers and Dornenburg ratios. The input vector of this model can be written as: B = [CH_4_/H_2_ C_2_H_2_/C_2_H_4_ C_2_H_4_/C_2_H_6_ C_2_H_2_/CH_4_ C_2_H_6_/CH_4_]^T^

The coordinates T_x_ and T_y_ in vector 6 are calculated, for each gas sample, as follows^18^:

6 $$T_{x} = \frac{1}{3A}\sum\limits_{i = 0}^{n - 1} {\left( {x_{i} + x_{i + 1} } \right)\left( {x_{i} y_{i + 1} - x_{i + 1} y_{i} } \right)} \,$$

7 $$T_{y} = \frac{1}{3A}\sum\limits_{i = 0}^{n - 1} {\left( {y_{i} + y_{i + 1} } \right)\left( {y_{i} x_{i + 1} - y_{i + 1} x_{i} } \right)} \,$$

A is the irregular triangle area given by:

8 $$A = \frac{1}{2}\sum\limits_{i = 0}^{n - 1} {\left( {x_{i} y_{i + 1} - x_{i + 1} y_{i} } \right)} \,$$

The coordinates x_i_ and y_i_(i = 0 to n − 1 with n = 3 is the number of gas in percentages) are computed as follows:

9 $${\text{x}}_{0} = \, \% {\text{CH}}_{{4}} {\text{cos}}\left( {\pi /{2}} \right)$$

10 $${\text{x}}_{{1}} = \, \% {\text{C}}_{{2}} {\text{H}}_{{4}} {\text{cos}}\left( {\pi /{2 } + \, \alpha } \right)$$

11 $${\text{x}}_{{2}} = \, \% {\text{C}}_{{2}} {\text{H}}_{{2}} {\text{cos}}\left( {\pi /{2 } + { 2}\alpha } \right)$$

12 $${\text{y}}_{0} = \, \% {\text{CH}}_{{4}} {\text{sin}}\left( {\pi /{2}} \right)$$

13 $${\text{y}}_{{1}} = \, \% {\text{C}}_{{2}} {\text{H}}_{{4}} {\text{sin}}(\pi /{2 } + \alpha )$$

14 $${\text{y}}_{{2}} = \, \% {\text{C}}_{{2}} {\text{H}}_{{2}} {\text{sin}}(\pi /{2 } + { 2}\alpha )$$

where α = 2π/3.

For the 7th Model, each sample, the coordinates P_x_ and P_y_ are computed by:

15 $$P_{x} = \frac{1}{6A}\sum\limits_{i = 0}^{n - 1} {\left( {x_{i} + x_{i + 1} } \right)\left( {x_{i} y_{i + 1} - x_{i + 1} y_{i} } \right)} \,$$

16 $$P_{y} = \frac{1}{3A}\sum\limits_{i = 0}^{n - 1} {\left( {y_{i} + y_{i + 1} } \right)\left( {y_{i} x_{i + 1} - y_{i + 1} x_{i} } \right)} \,$$

The pentagon surface Air given by:

17 $$A = \frac{1}{2}\sum\limits_{i = 0}^{n - 1} {\left( {x_{i} y_{i + 1} - x_{i + 1} y_{i} } \right)} \,$$

The parameters x_i_ and y_i_ (i = 0 to n − 1 with n = 5 is the gas number) are expressed by:

18 $${\text{x}}_{0} = \, \% {\text{H}}_{{2}} {\text{cos}}\left( {\pi /{2}} \right)$$

19 $${\text{x}}_{{1}} = \, \% {\text{C}}_{{2}} {\text{H}}_{{6}} {\text{cos}}\left( {\pi /{2 } + \, \alpha } \right)$$

20 $${\text{x}}_{{2}} = \, \% {\text{CH}}_{{4}} {\text{cos}}\left( {\pi /{2 } + { 2}\alpha } \right)$$

21 $${\text{x}}_{{3}} = \, \% {\text{C}}_{{2}} {\text{H}}_{{4}} {\text{cos}}\left( {\pi /{2 } + { 3}\alpha } \right)$$

22 $${\text{x}}_{{2}} = \, \% {\text{C}}_{{2}} {\text{H}}_{{2}} {\text{cos}}\left( {\pi /{2 } + { 4}\alpha } \right)$$

23 $${\text{y}}_{0} = \, \% {\text{H}}_{{2}} {\text{sin}}\left( {\pi /{2}} \right)$$

24 $${\text{y}}_{{1}} = \, \% {\text{C}}_{{2}} {\text{H}}_{{6}} {\text{sin}}(\pi /{2 } + \alpha )$$

25 $${\text{y}}_{{2}} = \, \% {\text{CH}}_{{4}} {\text{sin}}(\pi /{2 } + { 2}\alpha )$$

26 $${\text{y}}_{{3}} = \, \% {\text{C}}_{{2}} {\text{H}}_{{4}} {\text{sin}}(\pi /{2 } + { 3}\alpha )$$

27 $${\text{y}}_{{2}} = \, \% {\text{C}}_{{2}} {\text{H}}_{{2}} {\text{cos}}(\pi /{2 } + { 4}\alpha )$$

where α = 2π/5.

Other possible combinations of the above technique have also been proposed to give strong credibility to the obtained results. Two combinations are given below.

**Figure 1:**
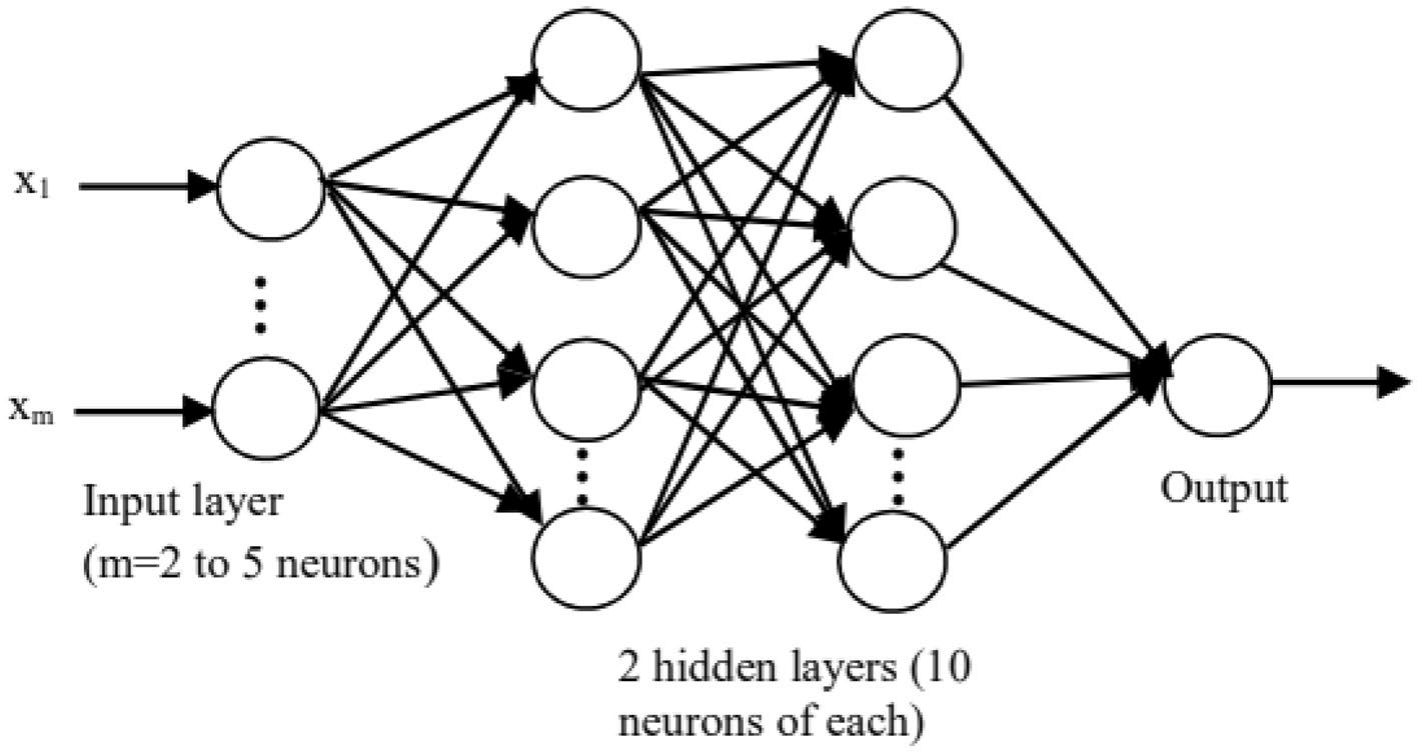
Topology of the MLP networks adopted in this study.

Using a large number of hidden layers is not recommended. Most classification standards problems use only one or at most two hidden layers^26^. Such ascertainments have been confirmed during our modeling. After several attempts, the best results have been achieved for two hidden layers of ten neurons of each. In addition, we have considered one output delivering, for each gas sample, a single fault (PD, D1, D2, T1, T2 or T3). Indeed, we introduce only the number of input neurons varying from 2 to 5 according to the elements number of the input vector. Thus, the topology of the multilayer neural networks we adopted in this work is shown in Fig. 1.

The weighing parameters of the layers are calculated using learning algorithms. Many algorithms are proposed in the literature^25,27^. We can find the supervised and unsupervised ones. In this paper, we are interested by the supervised ones. Supervised learning is done by introducing pairs of inputs and their desired outputs. This learning uses an optimization criterion allowing it to find optimal synaptic weights giving the desired behavior using random samples. Several learning techniques are adopted to readjust weights^27^. One of the most widespread algorithms is the “Back propagation”. Unfortunately, this algorithm suffers from the local minimum problem. A variant of the previous method consists of choosing an appropriate displacement to accelerate the convergence of the algorithm, which then leads to fast back propagation with momentum. There is another version called Robust Back propagation applicable in the stochastic case. In order to improve the choice of the direction to take in the weight space, we use to second-order optimization methods of the objective function specified by the Hessian.

Many learning algorithms, existing in the Matlab toolbox, have been tested. We have chosen three algorithms using the back-propagation of the quadratic error and only one using back-propagation of the gradient of the quadratic error^27^. This latter is between the response calculated by the network and the desired one.

In fact, back-propagation or gradient back-propagation algorithms are the most widely used models in supervised training. For this, several models are presented in the Matlab toolbox. These models are used to adjust the weights and biases satisfying the quadratic error or its gradient between the output value and the desired one reaches minimum value for every gas sample. The selected training algorithms are as follows:

- Levenberg–Marquardt back-propagation algorithm (trainlm).
- One-step secant back-propagation algorithm ( trainoss).
- Resilient backpropagation algorithm (trainrp).
- Scaled conjugate gradient back-propagation algorithm ( trainscg).

The choice of a specific algorithm depends on the input vector and the cost function. There is no theoretical method to select one algorithm versus another. In our case, we have used four training algorithms for different DGA techniques.

The activation function transforms the unbounded signal into a bounded one. This function is chosen non-decreasing monotonic. Increasing the input can only increase the output or keep it constant. Choosing a linear activation function makes calculation easier, but the neuron loses its robustness. A nonlinear activation function increases the network's ability to approximate complex functions. There are three categories of activation functions. The first one allows to distinguish between differentiable functions (sigmoid, tangent, hyperbolic) and non-differentiable ones (threshold function, thresholding). The second category concerns the functions that have significant values around zero, and significant values far from this one. The third ones deal with difference between positive functions and functions with zero mean (0 and 1 or – 1 and 1).

Five activation (transfer) functions are selected from the Matlab toolbox: softmax, radbas, purelin, logsig and poslin. Several attempts have been made to obtain the best diagnostic rates. The best combinations have been found when using softmax in the second hidden layer and purelin in the output layer. Indeed, the five aforementioned activation functions are applied only in the first hidden layer, maintaining, softmax and purelin for the second hidden layer and the output one respectively^30^.

The definitions of the selected activation functions are^30^:

- Normalized Exponential (softmax): It is a normalized function, which takes as input vector B of m elements and gives a vector of K strictly positive numbers whose sum equals 1. This function is defined by:
  28 $$f(x_{j} )\, = \,\frac{{e^{{x_{j} }} }}{{\sum\nolimits_{i = 1}^{m} {e^{{x_{i} }} } }}\,\,\,{\text{with}}\,\,\,j = 1, \, 2, \, \ldots ,m$$
- Radial Basic (radbas): it consists of the radial basic transfer function of Gaussian type, given by:
  29 $$f(r)\, = \,e^{{ - r^{2} }}$$
  r being a real replacing the Euclidean distance neuron-center^30^.
- Linear (purelin): this activation function is linear.
- Hyperbolic Tangent Sigmoid (tansig): It consists of the hyperbolic tangent sigmoid activation function defined by:
  30 $$f(x)\, = \,\frac{2}{{1\, + \,e^{ - 2x} }} - 1$$
- Rectified Linear Unit Layer (poslin). It is a rectified linear unit layer activation function. For an input value x, this function can be expressed by:
  31 $$f(x)\, = \,\left\{ {\begin{array}{*{20}c} {x,} & {x\, \ge \,0} \\ {0,} & {x < \,0} \\ \end{array} } \right.$$

The choice of the activation functions depends on the input data. These functions modify the data values from the input through the output. The combination of them leads to avoid the elimination of the information. So, it is necessary to proceed by some beginning choice of these functions. According to our experience, it is more convenient to make a combination of these functions from the input layer to the output one. In the hidden layer, a smoothie function is recommended to keep the information of the signal without truncation. This is very important for decision process and exploration of all the range of the input data.

## ANN-PSO models for insulating oil diagnostic by DGA

Our optimization problem aims to find the best solution, consisting of the global optimum, among a set of solutions belonging to the search space, by minimizing the MSE as function objective.

In fact, meta-heuristic optimization methods have the advantage of being adapted for a wide range of problems without major modification of their algorithms^28^. These methods are based on populations of solutions^29^. They are often inspired by natural processes, in particular by the theory of evolution in animal and insect societies which relate to evolutionary biology such as Genetic Algorithms (GA)^30^, or to the ethological theory such as Particle Swarm Optimization (PSO)^31^, Ant Colony Optimization (ACO)^32^, Social Spiders Optimization (SSO)^33,34^.

In our investigation, we opted for the PSO technique. To this end, we first present its principle, elements and parameters. We subsequently present the approach undertaken for optimizing the training of the ANN using PSO.

Particle swarm optimization is based on a homogeneous set of particles, initially arranged randomly. These particles move in the search space and each constitutes a potential solution. Each particle memorizes its best visited solution and communicates with the nearby particles. Thus, the particle will follow a trend based, on one hand, on its desire to return to its solution optimal, and, on other hand, on its mimicry with respect to the solutions found in its neighborhood on other hand. Indeed, from the local optima, the set of particles converges towards the optimal global solution of the treated problem^35^.

To be able to apply the PSO algorithm, one must define a search space of the particles and an objective function to be optimized. The principle is to move these particles to find the optimum. Each particle contains^36^:

- A position characterized by its coordinates in the definition space of the objective function: ${X}_{i}=({X}_{i1},\dots ,{X}_{ij},\dots ,{X}_{ik})$
- A velocity allowing the particle to change position during the iterations according to its best neighborhood, its best position, and its previous position: ${V}_{i}={(V}_{i1},\dots ,{V}_{ij},\dots ,{V}_{ik})$
- A neighbourhood constituted by the set of particles directly interacting on the particle, in particular the one having the best value of the objective function.

At any moment, each particle knows:

- Its best visited position *P*_*i*_*(t)* through its coordinates and the value of the objective function;
- The position of the best neighbour of the swarm *g*_*i*_*(t)* which corresponds to the optimal scheduling;
- The value assigned to the objective function f (*P*_*i*_*(t)*) at each iteration following the comparison between the value of this function given by the current particle and the optimal one.

The particle swarm algorithm is based on:

- Population size: According to Van den Bergh and Engelbrecht^35^, increasing swarm size slightly improves the optimal value. Eberhart and Shi^36^ illustrated that population size has a minimal effect on the performance of the EP method. The same observation was made by Nezhad and his colleagues^37^. In our investigation, various population sizes ***N***_*p*_, namely ***N***_*p*_ = 40, 80, 100 and 120.
- Initialization of position and velocity: Before generating the population of particles, it is necessary to define the search space for them and place them randomly according to a uniform distribution. In a d-dimensional search space, the particle i of the swarm is modelled by its position vector X_i_ according to Eq. (33), and by its velocity vector *V*_*i*_ according to Eq. (32)^38^.
- Position and velocity update: The quality of the particle position is determined by the value of the objective function. Along its path, this particle memorizes its best position, which we note *p*_*best*_ = (*p*_*i1*_*,…, p*_*i2*_*,…, p*_*id*_). Furthermore, the best position found for its neighbouring particles is *g*_*best*_ = (*g*_*i1*_*,…, g*_*i2*_*,…, g*_*id*_). At each iteration, the particles update their positions and velocities taking into account their best positions and those of its neighbourhood^39^.

The new velocity is calculated by Ref.^41^:

32 $${V}_{i}\left(k+1\right)={wV}_{i}\left(k\right)+{{c}_{1}r}_{1}\left(k\right)\left({p}_{best}\left(k\right)-{X}_{i}\left(k\right)\right)+{{c}_{2}r}_{2}\left(k\right)\left({g}_{best}\left(k\right)-{X}_{i}\left(k\right)\right)$$

Therefore, the new position velocity is calculated as follows^40^:

33 $${X}_{i}\left(k+1\right)={X}_{i}\left(k\right)+{V}_{i}\left(k+1\right)$$

*X*_*i*_*(k), X*_*i*_*(k* + *1)*: the positions of the *P*_*i*_ particle at iteration k and k + 1 respectively ; *V*_*i*_*(k), V*_*i*_*(k* + *1)*: the velocities the *P*_*i*_ particle at iteration k and k + 1 respectively; *p*_*best*_*(k* + *1)*: the best position obtained by the *P*_*i*_ particle at iteration *k* + *1*; *g*_*best*_*(k* + *1)*: the best position obtained by the swarm at iteration *k* + *1*; *c*_*1*_* et c*_*2*_: constants representing the acceleration coefficients; *r*_*1*_* et r*_*2*_: random numbers; *w(k)*: inertial weight.

## Methods

In the previous ANN presentation, each neural network delivers the best classification rate by minimizing the root mean quadratic error, MSE, estimated from the computed outputs and the desired ones, as a function of synaptic weights and biases.

The biases consist of b_h1_, b_h2_ and b_o_. b_h1_ and b_h2_ are vectors of 10 elements each. The first is presented to the first hidden layer, and the second to the second hidden layer. b_o_ is an additional scalar added to the output layer.

The synaptic weights consist of w_i_ between the input layer and the first hidden layer, w_h_ inter-hidden layers, and finally w_o_ between the second hidden layer and the output one.

w_i_ is a matrix having a number of rows equal to that of input vector elements (m varying from 2 to 5). Moreover, the number of columns of w_i_ is equal to the number of neurons of the first hidden layer, i.e. q = 10. In this hidden layer, we applied an activation function, f_1_. The five previous functions (softmax, radbas, purelin, tansig and poslin) have been tested for this latter.

w_h_ linking the first hidden layer to the second, is also a square matrix of dimension qxq (i.e. 10 × 10). The activation function, denoted f_2_, applied in the second hidden layer, is sotfmax. As previously indicated, this activation function has been kept unchanged throughout the diagnostic process.

Likewise, w_o_ connecting the second hidden layer and the output layer, is also a matrix of dimension 1 × 10 (i.e. a vector of 10 elements). The activation function, denoted f_3_, which has been adopted in the output layer is purelin. This latter has also kept unchangeable. In such conditions, the output value is given by:

34 $$Y= {f}_{3}(\sum {w}_{o}*{f}_{2}( \sum {w}_{h}*{f}_{1}(\sum {w}_{i}*B)+{b}_{h1})+{b}_{h2})+{b}_{o}$$

f_1_, f_2_ and f_3_ are the activation functions, b_h1_, b_h2_ and b_s_ the biases and B the input vector.

The mean value of the mean squared error has been used as the objective function giving by the following expression:

35 $$MSE={\frac{1}{TS}\sum_{PT}{(Y}_{d}-Y)}^{2}$$

where TS is the total number of training samples (equal to 321), Y is the computed network output, and Y_d_ is the desired output.

Our contribution in this paper that we have proposed the new model for DGA by combining the artificial neural network and particle swarm optimization algorithm which gives significant results with very high accuracy. This hybridization has never been developed until now.

Taking into consideration the mathematical calculation of the PSO model in the previous section, the convergence of the PSO towards the global optimum depends on the following parameters:

- Inertia factor: The inertia factor w allows controlling the impact of previous velocities on the actual one^41^.
  - If w <  < 1, rapid changes of direction are possible; little of the previous velocity is preserved;
  - If w = 0, the particle moves in each step without knowledge of the previous velocity; the concept of velocity is completely lost;
  - If w > 1, the particles barely change their direction, which results a great area of exploration and a hesitation against convergence towards the optimum.
- Acceleration coefficients *c*_*1*_ et *c*_*2*_*:* The constant c_1_ affects the acceleration of the particle towards its best performance (cognitive behaviour of the particle). Otherwise, c_2_ allows the particle to accelerate towards the Global Best (social ability of the particle)^42^. These constants belong to the interval [0; 2]^39^. In our investigation, *c*_*1*_ equals 2 and *c*_*2*_ equals 1.
- Random numbers *r*_*1*_* et r*_*2*_*:* At each iteration, the two parameters *r*_*1*_*(k)* and *r*_*2*_*(k)* are generated randomly in the interval [0; 1] by a uniform distribution^41^.
- Stopping criterion: In order to converge towards the global optimal solution, different stopping criteria can be selected. The most commonly used consist of^42^:
  - Static criterion: it is generally based on the maximum number of iterations;
  - Dynamic criterion: it refers to the stagnation of velocity.

The static criterion has been adopted in our study. The number of iterations has been fixed to 10,000.

A high value of the inertia factor facilitates exploration (the search for new sectors). A low value facilitates exploitation (favoring the current sector of research)^42^. Better convergence, providing the balance between exploration and exploitation. Furthermore, it is possible to vary this factor during the iterations according to Eq. (36). Good results were obtained for an increase value from 0.4 to 0.9.

36 $$w\left(k\right)={w}_{min}+\left({w}_{max}-{w}_{min}\right) \left(\frac{k}{{max}_{iter}}\right)$$

*w*_*max*_*: the* maximum value of w (= 0.9); *w*_*min*_: the minimum value of w (= 0.4); *iter: the* current iteration; *max*_*iter*_: the maximum number of iterations.

Before applying the PSO to optimize the ANN training, first, it is necessary to choose the architecture (topology) of the ANN (of two hidden layers with 10 neurons for each layer), the training algorithms, the activation functions, PSO parameters (population sizes, maximum number of iterations, variables to optimize, etc.), the database as well as the number of samples reserved for training and testing. Next, it is necessary to determine the objective function to be optimized. This function consists of the mean square error given by Eq. (35).

The PSO algorithm is employed in training the ANN to determine the set of parameters *w* and *b*. The total number (corresponding to search space dimension) of these parameters can be determined by the following equation:

37 $${\varvec{N}}=\left({{\varvec{s}}}_{{\varvec{i}}}\boldsymbol{*}{\varvec{q}}+{{\varvec{b}}}_{{\varvec{h}}1}\right)+\left({\varvec{q}}\boldsymbol{*}{\varvec{q}}+{{\varvec{b}}}_{{\varvec{h}}2}\right)+{\varvec{q}}+\boldsymbol{ }{{\varvec{b}}}_{{\varvec{o}}}$$

*s*_*i*_: input vector dimension.

The execution of the ANN-PSO algorithm is carried out in accordance with the following steps:

- **Step 1:** Upload the data training set and the data test one.
- **Step 2:** Define the architecture of ANN: number of hidden layers, neurons number, train algorithm and activation function.
- **Step 3:** Determine the PSO algorithm parameters and randomly generate *p* particles.Each one contains*N* values of weights and biases, and generate, then after, *N*_*p*_ANN models.
- **Step 4:** Train the *N*_*p*_ ANN and calculate objective functionMSE(*p* values) using weighs and biases generated by PSO.
- **Step 5:** Select the best solution and update it if it is different from the previous iteration.
- **Step6:** Check the criterium iteration.
- **Step 7:** If the condition of step 6 is not verified, return to step 4 with updating the particles velocities and positions. Else, if the condition of step 6 is verified, the optimal parameters are used to test the ANN.

We have compared in the same conditions, the performance of the two algorithms. Indeed, the same multilayer topology used has been kept for this part. Also, we have adopted the input vector 9 (of the coordinates of the two centers of mass of triangle 1 and pentagon I of Duval) offering the best classification performance of 90% for neural network. This result was obtained using trainlm as training algorithm, and poslin, softmax and purelin as activation functions respectively in the first hidden layer, the second hidden one and in the output one. This training algorithm-activation functions combination has been kept in the second algorithm, in order to further improve the fault classification rate. Also, we have considered the same database of 481 samples including 321 samples (set of 66%) for training and 160 (set of 33%) for testing, the same gases (H_2_, CH_4_, C_2_H_2_, C_2_H_4_ and C_2_H_6_), and the same defects (PD, D1, D2, T, T2, and T3).

In order to ensure the convergence of the objective function (the mean square error) towards an optimum, four population sizes of the particle swarm have been adopted, namely 40, 80, 100 and 120.

The search space dimension, corresponding to the number of parameters *w* and *b*,has been fond equal to *N* = 171. The parameters of the PSO algorithm have been set as described in the Table 1.

**Table 1:** PSO algorithm parameters values.

Parmeter	Descriotion	Value
*c*_*1*_	Particle coefficient	2
*c*_*2*_	Swarm coefficient	1
*iter_max*	Maximum iteration	10,000
*N*	Search space dimension	171

## Results

For ANN results, 180 programs of neural networks have been developed from the nine input vectors, four training algorithms and five activation functions. The database contains 481 samples. For each network (model), 66% (i.e. 321 gas samples) have been selected for training and the rest (160 samples) for the test.

The performance of each neural network has been evaluated in terms of classification or diagnosis rate. For this, the number of iterations adopted is 1000. Each neural network has been executed 50 times, and the best diagnosis rate has been recorded.

**Figure 2:**
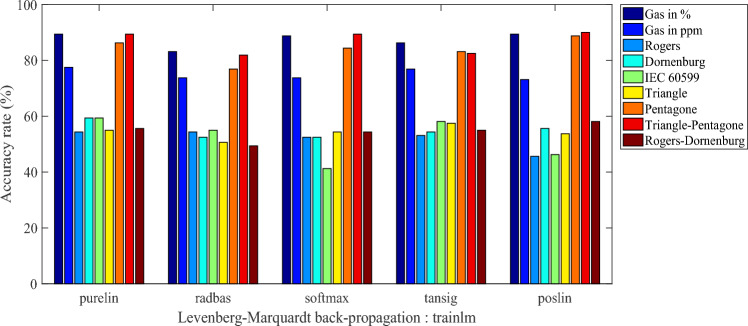
Accuracy rate-activation function of the first hidden layer, for trainlm training algorithm.

**Figure 3:**
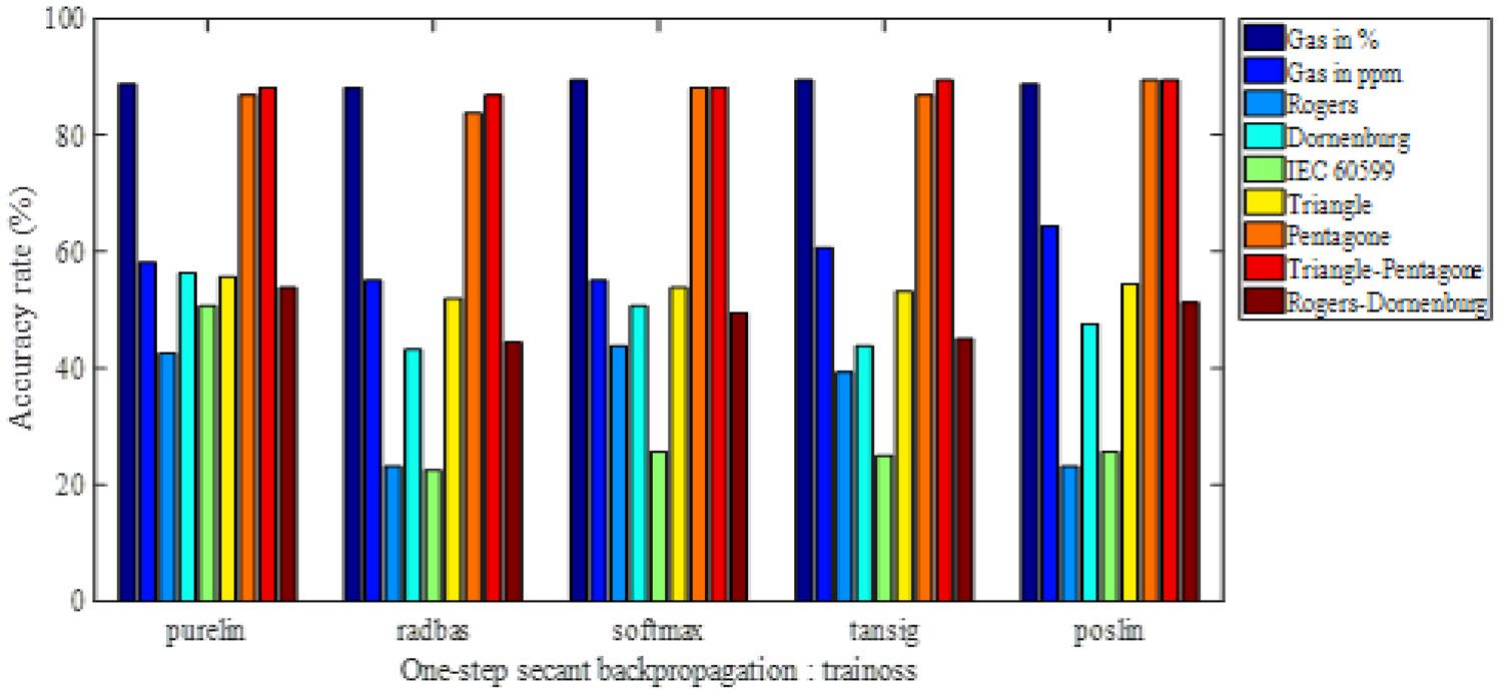
Accuracy rate-activation function of the first hidden layer, for trainoss training algorithm**.**

**Figure 4:**
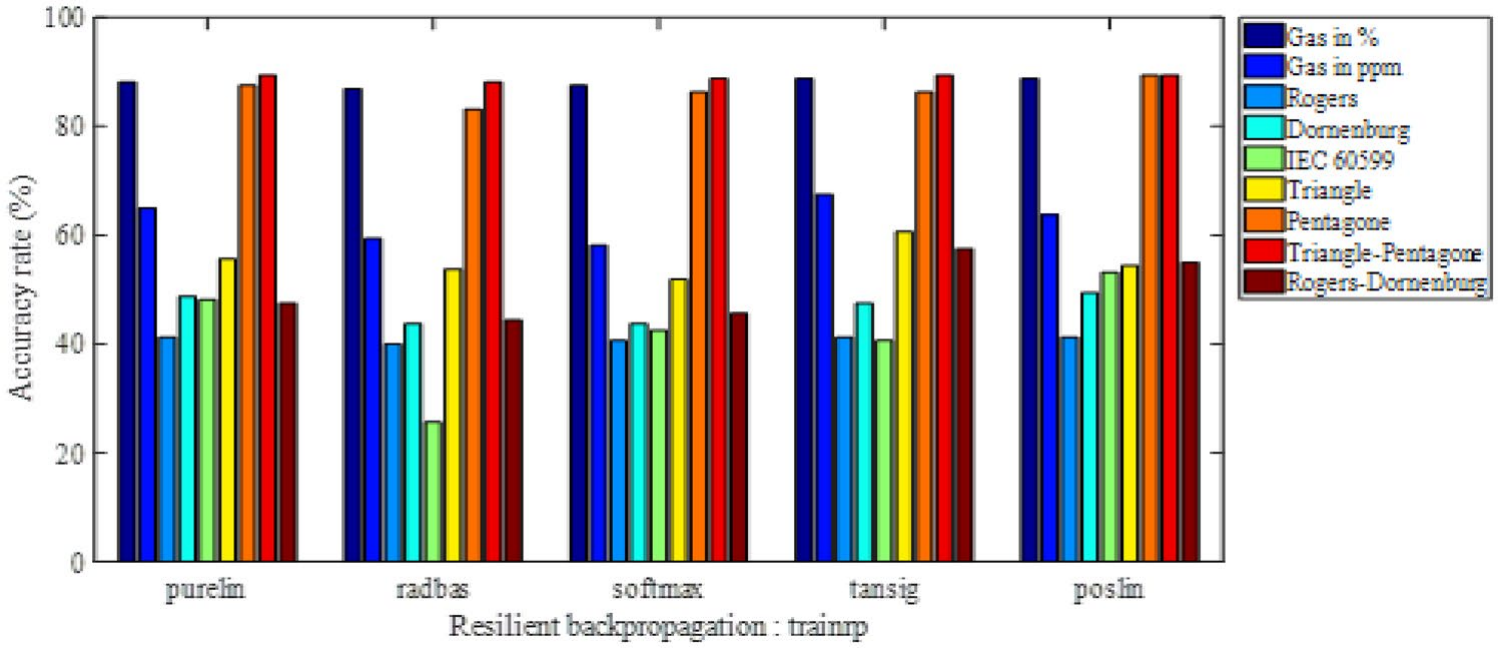
Accuracy rate-activation function of the first hidden layer, for trainrp training algorithm.

**Figure 5:**
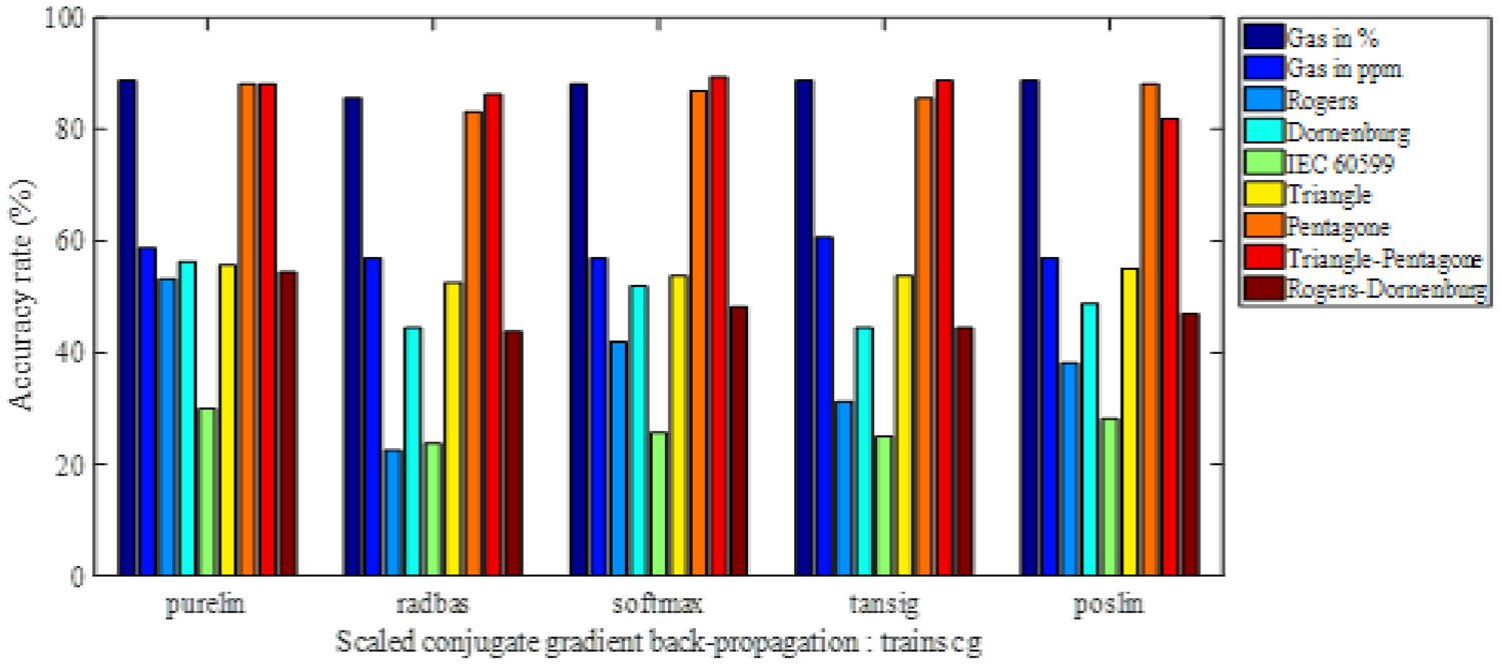
Accuracy rate-activation function of the first hidden layer, for trainscg training algorithm.

The obtained results are presented as histograms of Figs. 2, 3, 4 and 5. These figures illustrate the different diagnostic rates as a function of the activation function of the first hidden layer, for different training algorithms trainlm, trainrp, trainoss and trainscg, respectively. Note that the softmax and purelin have been used as activation functions in the second hidden layer and the output one, respectively.

In fact, the four figures were drawn using twenty tables (five tables per figure). To avoid burdening the manuscript, we prefer to present only one Table 2 employing purlin algorithm for the input layer (for the first algorithm Purlin of Fig. 2). Such tables provide more details of the results in terms of activation functions for different input layers, accuracies and input vectors. This allows a better comparison between the considered models.

**Table 2:** Details for Purelin algorithm by ANN in Fig. 2.

Activation functions			Accuracy Levenberg–Marquardt (%)	Inpit vectors
Input layer	Hiden layer	Output layer		
Purelin linear transfer	Softmax normalized exponnetial	Purelin linear transfer	89.38	Five gazes in pourcentage
			77.50	Five gazes in ppm
			54.38	Rogers's Four ratio
			59.38	Dornenburg"s Four ratio
			59.38	LCEI 60,599 Three Ration
			55.00	Duval's Triangle
			86.25	Duval's Penatgone
			89.38	Duval's Triangle & Pentagone
			55.63	Rogers &Dornenburg's ratio

We are interested, through the results of Figs. 2, 3, 4 and 5, to determine the highest classification rate for each model. The input vectors can be classified in decreasing order of the classification rate (i.e. from best to bad) as follows:

- 8th Model gives a maximum rate of 90%, i.e. 144 faults well classified on 160 (reserved for the test).
- 2nd Model gives a maximum rate of 89.375% corresponding to 143 well-classified faults.
- 1rt Model gives a maximum rate of 77.5%, i.e. 124 well-classified faults.
- 6th Model gives a maximum rate of 60.625% is 97 well-classified faults.
- 3rd Model and 5th model of IEC 60,599 ratios have given a maximum rate of 59.375%, so 95 are well-classified faults.
- 9th Model has given a maximum rate of 58.125%, i.e., 93 well-classified faults.
- 4th Model gives a maximum rate of 54.375%, corresponding to 87 well-classified faults.

In order to provide all information of such classification, we present in the Table 3 the accuracy rate in descending order (from best to bad) for all input vectors with the activation functions of the first hidden layer as well as the training algorithms. Note that the activation functions in the second hidden layer and the output one are softmax and purelin respectively.

**Table 3:** Accuracy rates in descending order with activation functions of the first hidden layer and training algorithms.

Vector	Accuracy rate (%)	Classified faults/160	Training algorithm	Activation function of the 1st hidden layer
8	90	144	Trainlm	Poslin
2	89.375	143	Trainlm	Purelinor poslin
7	89.375	143	Trainoss	Poslin
1	77.500	124	Trainlm	Purelin
6	60.625	97	Trainrp	Tansig
3	59.375	95	Trainlm	Purelin
5	59.375	95	Trainlm	Purelin
9	58.125	93	Trainlm	Poslin
4	54.375	87	Trainlm	Purelinor radbas

**Figure 6:**
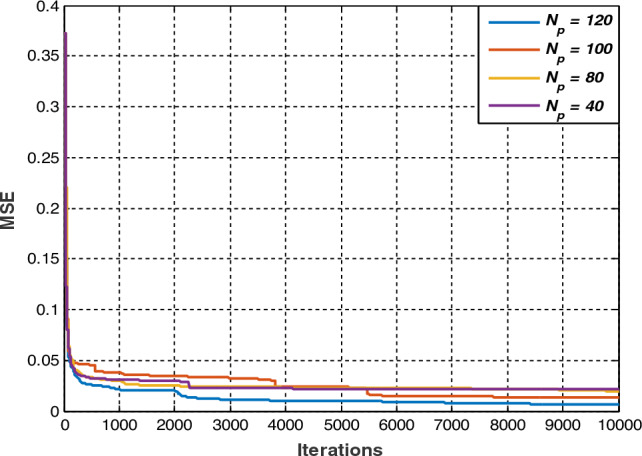
Variation of MSE as a function of the number of iterations, for different population sizes.

For PSO results, the variation of the objective function, consisting of the mean squared error (MSE), as a function of the number of iterations, for different population sizes is presented in Fig. 6.

Depending on the population size, the minimum mean square error, the precision rate and the number of well-classified faults, are summarized in Table 4.

**Table 4:** Diagnosis accuracy with several population sizes.

Population size	40	80	100	120
Global MSE	0.0216	0.0197	0.0173	0.0066
Accuracy rate (%)	96.25	98.125	98.750	99.375
Number of well-classified samples/160	154	157	158	159

## Discussion

The training algorithm trainlm contributed in the elaboration of these results by approximately 78%, since it has been used 7 times on 9. Otherwise, the activation functions purelin and poslin have participated in these results of about 56% (5 on 9) and 44% (4 on 9) respectively. Indeed, for such classification type, it is recommended to use trainlm as training algorithm, purelin or poslinas as activation function in the first hidden layer, softmax for the second hidden one and purelin in the output one.

The best classification rate of 90%, i.e. 144 well-classified faults on 160), has been obtained by presenting the input vector consisting of the coordinates of the two centers of mass of the triangle and the pentagon Duval, using trainlm as training algorithm and poslin, softmax and purelin as activation functions in the first hidden, the second hidden and the output layers respectively. This accuracy rate will be improved, in the same conditions, using particle swarm optimization (PSO) technique.

For a given population size, and over the iterations, Fig. 6 shows that the MSE (objective function) decreases abruptly from 0 to 120 iterations, and slowly elsewhere, tending practically towards a constant level. This latter is called minimum MSE which could represent the global MSE. It changes from one population to another, as illustrated in Table 3. In fact, with the progressive increase in the population size from 40 to 120, the global MSE slightly decreases from 0.0216 to 0.0066, while the classification rate and therefore the number of well-classified faults slightly increases from 154 to 159 reserved for the test. Indeed, the 120-population size allows obtaining 159 well-classified faults out of 160 (reserved for the test) with an accuracy rate of 99.375% against 90% when using ANN alone.

## Conclusion

In this investigation, we have developed intelligent techniques using multilayer feed-forward ANN-based models for fault detection in an oil-immersed power transformer, by analysis dissolved gases. Nine input vectors have been used. Otherwise, each hidden layer contains ten neurons. Finally, the output having only one neuron delivers a single fault for each gas sample.

Using back-propagation, four training algorithms have been chosen, namely trainlm, trainoss, trainrp and trainscg. In addition, five activation functions, consisting in softmax, radbas, purelin, tansig and poslin, have been selected. These functions have been applied for the first hidden layer, while softmax was maintained for the second hidden layer, and purelin for the output layer. The used database contains 481 samples of which 321 have been selected for training and the rest (160 samples) for testing. Inspired by IEC and IEEE standards, six faults, consisting in PD, D1, D2, T1, T2, and T3, have been adopted. The best classification rate of 90% (i.e. 144 well-classified faults out of 160) has been obtained when using the eighth input vector (formed from the coordinates of the two centers of mass of triangle 1 and pentagon I of Duval) and applying trainlm as learning algorithm.

In order to further improve the best classification rate, the corresponding multilayer network has been optimized using particle swarm technique for various population sizes, namely 40, 80, 100 and 120. The mean square error (MSE) represents the objective function to be minimized for 10,000 iterations. Obviously, the same database with the same number of samples for training and testing, and the same faults has been kept. For a given population size, the mean square error (the objective function) decreases abruptly for iterations ranging from 0 to 120, and slowly elsewhere, tending towards a constant level representing the minimum mean square error (MMSE). Furthermore, the gradual increase in the population size from 40 to 120 results in a slight decrease in the minimum squared error from 0.0216 to 0.0066, and a slight increase in the classification rate of 96.250 (corresponding to 154 faults well classified out of 160) at 99.375% (with 159 well classified faults). In other words, the best fault classification rate of 99.375% has been obtained for 120 population size. In these conditions, the ANN-PSO algorithm was able to detect 159 faults out of 160 reserved for the test.

Finally, our contribution in the paper is to present a new approach by combining learning and particle swarm optimization in dissolved gas analysis field. We have demonstrated that this technique is leading to significant results comparing them to the existing ones in the previous research. We have obtained high level of decision about the quality of the transformer oil by using different methods according to IEC and IEEE standards.

In high voltage distribution, this new model facilitates the maintenance process and avoids transformer failure. It gives us the instantaneous decision about the characteristics of the failure and time life of the transformer. This latter may include smart sensors linked to digital process unit with supervisory and data acquisition system. The advantage to use this model is to control in real time the process by minimum time calculation of the decision. It leads time saving and minimum cost.

It is worth noting that the best results are obtained with the ANN-PSO model. This hybridization is the key to reach this objective. The choice of the architecture of the neural network was the crucial phase in our study. Also, the combination of different training algorithms and activation functions allows obtaining the best model with several tests. The ANN-PSO model needs many calculations to have the convergence of the algorithm. However, it is necessary to match the obtained models to the corresponding oil analysis technique. As input vector, the graphical methods of Duval give decision with best score.

In order to use our technique in other field, it is necessary to adapt the architecture of the neural network to the problem. It means that we can choose the input layer and the output layer according to the proposed problem. After that, the number of the hidden layer can be fixed according to the accuracy of the result. Finally, it is important to have a deep knowledge of the application that we want to use the model developed in this paper.

## Data Availability

The datasets used and/or analysed during the current study available from the corresponding author on reasonable request.
